# Prognostic factors of survival in patients treated with nab-paclitaxel plus gemcitabine regimen for advanced or metastatic pancreatic cancer: A single institutional experience

**DOI:** 10.18632/oncotarget.3143

**Published:** 2015-03-10

**Authors:** Giovanni Lo Re, Davide A. Santeufemia, Luisa Foltran, Ettore Bidoli, Stefano M.M. Basso, Franco Lumachi

**Affiliations:** ^1^ Oncology Unit, S. Maria degli Angeli Hospital, 33170 Pordenone, Italy; ^2^ Epidemiology Unit, Centro di Riferimento Oncologico (CRO), 33081 Aviano, Pordenone, Italy; ^3^ Surgery 2, S. Maria degli Angeli Hospital, 33170 Pordenone, Italy; ^4^ Department of Surgery, Oncology and Gastroenterology, University of Padua, School of Medicine, 35128 Padova, Italy

**Keywords:** advanced pancreatic cancer, metastasis, nab-paclitaxel, gemcitabine, prognostic factors

## Abstract

**Purpose:**

The objectives of this study were to evaluate the effectiveness of nab-paclitaxel plus gemcitabine (NAB-P/GEM) regimen in an unselected population of patients with advanced inoperable or metastatic pancreatic cancer (PC), and to identify the prognostic factors influencing overall survival (OS).

**Experimental design:**

Patients with age < 85 years, ECOG-performance status (PS) < 3, and adequate renal, hepatic and hematologic function were eligible. NAB-P (125 mg/m2) and GEM (1000 mg/m2) day 1,8,15 every 4 weeks were employed for 3–6 cycles or until highest response.

**Results:**

Overall, 147 cycles (median 4, range 1–11 cycles) were administered on thirty-seven consecutive patients (median 66 years old, range 40–82) treated. The median overall progression-free survival and OS were 6.2 and 9.2 months, respectively. The G 3–4 dose-limiting toxicity were neutropenia (20.7%), severe anemia (17.2%), and cardiovascular toxicity (10.3%). PS, number of cycles, baseline CA 19–9 and LDH serum levels, were found to be significantly related to OS. The multivariate analysis showed that both number of cycles (HR = 9.14, 95% CI 1.84–45.50, *p* = 0.001) and PS (HR = 13.18, 95% CI 2.73–63.71, *p* = 0.001) were independently associated with OS.

**Conclusion:**

NAB-P/GEM regimen should be used in all patients with advanced or metastatic PC, with the exception of those with serious contraindications to chemotherapy, such as severe renal or hepatic impairment or major cardiovascular diseases.

## INTRODUCTION

Pancreatic cancer (PC) is an aggressive tumor that accounts for approximately 3% of all cancers, whose incidence is increasing [1]. It represents the fourth leading cause of cancer death in the United States in both gender. PC remains a chemoresistant malignancy and the nucleoside analogue gemcitabine, which has represented for years the standard first-line therapy for patients with PC, provides limited clinical benefits, especially in advanced or metastatic disease [2, 3]. The prognosis of patients with PC is poor and their 5-year overall survival (OS) rate is only 5%, as approximately 80% of patients are diagnosed when the disease has already given regional or distant metastases [1, 4]. Several alternative drugs have been tested, alone or in combination with gemcitabine, including oxaliplatin, capecitabine, cisplatin, 5-fluorouracil (5FU), erlotinib and bevacizumab, but none has significantly improved OS [5–10].

Two four-drug regimens, named PEFG (cisplatin, epirubicin, fluorouracil, gemcitabine) and FOLFIRINOX (folinic acid, 5FU, irinotecan, oxaliplatin), have been shown to give better results towards gemcitabine alone [11, 12]. More recently, a multicentric study comparing nab-paclitaxel (nanoparticle albumin-bound paclitaxel) plus gemcitabine (NAB-P/GEM) versus gemcitabine alone, reported significant survival benefit (1-year OS rate 35% vs. 22%) of this new regimen, offering another possibility in the management of patents with PC [13]. However, the exclusion criteria adopted in the selection of patients (i.e., Karnofsky performance-status score < 70 on a scale from zero to 100, no previous chemotherapy for metastatic disease), restrict the use of this treatment protocol.

The purposes of the present study were (i) to evaluate the effectiveness of NAB-P/GEM regimen in an unselected population of patients with advanced inoperable or metastatic PC and (ii) to identify the prognostic factors influencing OS.

## RESULTS

### Patient characteristics

A series of 37 consecutive patients (20 males, 17 females; median 66 years old, range 40–82) with locally advanced unresectable primary tumor (*N* = 6, 16.2%) or metastatic (*N* = 31, 83.8%) PC were enrolled in the study. The local institutional ethics committee obtained written informed consent from all participants before therapy started and the study had full ethical approval. All patients were evaluated according to Eastern Cooperative Oncology Group performance status (ECOG-PS) protocol that uses a scale from 0 to 5, with higher score indicating illness or a very poor PS [14].

Patients underwent baseline standard hematological check-up, including complete blood cell count, bilirubin, creatinine, alanine aminotransferase (ALT), alkaline phosphatase (ALP), lactate dehydrogenase (LDH), which were assayed using laboratory routine methods. Serum carbohydrate antigen (CA) 19–9 was measured using a commercially available immunoassay by automated heterogeneous chemiluminescent immunoassays that use N-(aminobutyl)-N-(ethylisoluminol) as luminescence substrate (Maglumi, Shenzen New Industries Biomedical Engineering, SNIBE, Shenzen, China).

Overall, the median baseline CA 19–9 level was 1734 U/mL (range 2–120,000 U/mL), while the median values of hemoglobin, white blood cells (WBC), ALP and LDH were 12 g/dL, 7230/μL, 136 U/L and 205 U/L, respectively.

### Toxicity and antitumor activity

**Figure 1:**
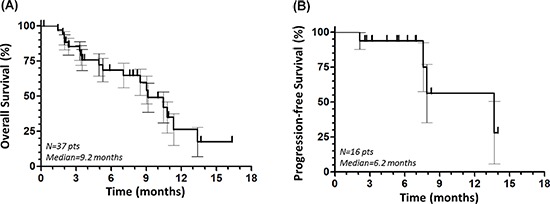
Overall survival **(A)** and progression-free survival **(B)** of the overall population.

We obtained complete response in three (10.3%) and partial response in four (13.8%) out of 29 evaluable patients, whilst 16 (55.2%) and six (20.7%) patients had stable disease or progressive disease, respectively. The overall objective response rate and disease control rate were 19% (95% CI, 8%–30%) and 62% (95% CI, 55%–69%), respectively, and the median duration of response was 8 months (range 1–9 months). The median overall progression-free survival (PFS) and OS were 6.2 and 9.2 months, respectively (Figure 1A–1B). The grade (G) 3–4 dose-limiting toxicity were neutropenia in six (20.7%), severe anemia in five (17.2%), thrombocytopenia in two (6.9%), signs and symptoms of neurological and cardiovascular toxicity in three (10.3%) and one (3.4%) patients, respectively. One more patient (3.4%) complained of fatigue. Eight (21.6%) patients were not evaluable for early suspension.

**Figure 2:**
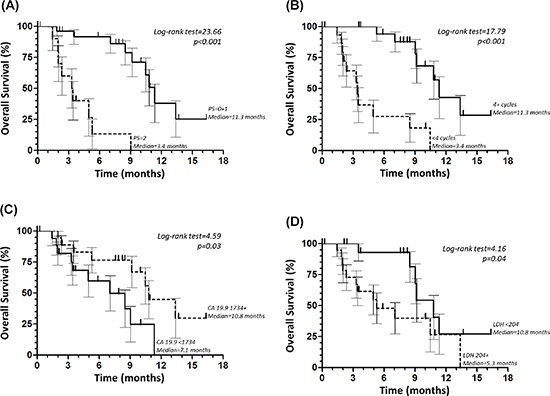
Overall survival according to Eastern Cooperative Oncology Group performance status (PS) **(A)** number of cycles **(B)** baseline carbohydrate antigen (CA) 19–9 **(C)** and lactate dehydrogenase (LDH) **(D)** serum levels.

The results of the univariate analysis are shown in Table 1. ECOG-PS (0–1 vs. 2), number of cycles (< 4 vs. ≥ 4), baseline CA 19–9 (< 1734 vs. ≥ 1734 U/mL) and LDH (< 204 vs. ≥ 204 U/L) serum levels, were found to be significantly related to OS (Figure 2A–2D). The multivariate analysis showed that both number of cycles (HR = 9.14, 95% CI 1.84–45.50, *p* = 0.001 for < 4 vs. ≥ 4 cycles) and PS (HR = 13.18, 95% CI 2.73–63.71, *p* = 0.001 for 2 vs. 0–1 ECOG-PS) were independently associated with OS.

**Table 1:** Association between selected prognostic factors and overall survival The hazard ratio (HR) is adjusted for all factors statistically significant in Kaplan-Meier analysis.

Parameter	Median OS (months)	Log-rank	*p*	HR	95% CI	*p*
Age:		0.48	0.49	-	-	
< 66 years	10.5					
≥ 66 years	9.0					
Gender:		0.13	0.72	-	-	
Male	10.8					
Female	9.2					
ECOG-PS		23.66	< 0.001			
0–1	11.3			1	2.73–63.71	0.001
2	3.4			13.18		
No. of cycles		17.79	< 0.001			
≥ 4	11.3			1	1.84–45.50	0.001
< 4	3.4			9.14		
Hemoglobin		0.09	0.76	-	-	
< 12 g/dL	9.0					
≥ 12 g/dL	10.5					
WBC		0.09	0.76	-	-	
< 7230/μL	9.2					
≥ 7230/μL	10.8					
ALP		0.27	1.24	-	-	
< 136 U/L	10.5					
≥ 136 U/L	7.1					
LDH		4.16	0.04			
< 204 U/L	10.8			1	0.27–4.41	0.91
≥ 204 U/L	5.3			1.09		
CA 19–9		4.59	0.03			
< 1734 U/mL	10.8			1	0.29–3.35	0.99
≥ 1734 U/mL	7.1			0.99		

OS, overall survival; p, *p*-value; HR, hazard ratio; 95% CI, 95% confidence interval; ECOG-PS, Eastern Cooperative Oncology Group performance status; WBC, white blood cells; ALP, alkaline phosphatase; LDH, lactate dehydrogenase; carbohydrate antigen (CA) 19–9.

## DISCUSSION

Pancreatic cancer remains a very serious disease, characterized by a poor prognosis, for which a multidrug therapy is usually more effective than monotherapy. In a group of patients with PC who underwent FOLFIRINOX regimen *versus* gemcitabine alone, the median OS was 11.1 vs. 6.8 months (HR = 0.57, *p* < 0.001) and the median PFS was 6.4 vs. 3.3 months (HR = 0.47, *p* < 0.001) compared to the control group [12, 15]. Unfortunately, FOLFIRINOX increases gastrointestinal and neurosensorial toxicity, thus reducing the quality of life parameters compared to gemcitabine regimen alone in patients with metastatic PC [15]. Moreover, the exclusion criteria of FOLFIRINOX protocol, such as age ≥ 76 years, ECOG-PS ≥ 2, and site of primary tumor, may partially justify the longer OS, thus limiting its use in unselected population of patients with PC [16].

Another effective regimen is NAB-P/GEM that demonstrates superiority on response rate and PFS over gemcitabine [13]. Nab-paclitaxel was initially developed to avoid hypersensitivity phenomena related to solvents such as polyethylated castor oil (Cremophor EL, BASF, Ludwigshafen, Germany) used as vehicle [17]. In PC with dense stroma and high levels of secreting protein acidic and rich in cysteine, the efficacy of NAB-P is higher because the albumin-binding protein sequestrates NAB-P, which concentrates within the tumor tissue [18]. It also synergize with gemcitabine, which increases by 2.8 times the intratumoral concentration of the drug [19]. NAB-P/GEM should also be considered as salvage therapy after ineffective first-line FOLFIRINOX treatment [20].

Using The Quality-Adjusted Time Without Symptoms or Toxicities (Q-TWiST) methodology it has been shown that patients treated with NAB-P/GEM have a significant gain in the quality-adjusted survival, when compared to those treated with gemcitabine alone [21]. A meta-analysis on studies of patients with metastatic PC treated with single-agent or combination chemotherapy regimens, including NAB-P/GEM and FOLFIRINOX, showed the superiority of multidrug protocols *versus* gemcitabine alone and the substantial equivalence between NAB-P/GEM and FOLFIRINOX [4]. Another review confirmed that both regimens should be considered the treatment of choice as first-line chemotherapy in patients with metastatic PC [22]. The results of our study confirm the effectiveness of the protocol NAB-P/GEM (PFS = 6.2 months, OS = 9.2 months) also in a population of unselected patients with advanced or metastatic PC, and the multivariate analysis showed that both PS and number of cycles of therapy were independently associated with OS. This suggests that even patients with PS < 2 may tolerate a high number of cycles of therapy, which could enable them to prolong OS.

## METHODS

### Patient selection

Inclusion criteria were the following: (i) age < 85 years, (ii) ECOG-PS < 3, (iii) histological diagnosis of pancreatic non-neuroendocrine adenocarcinoma, (iv) metastatic PC on ^18^F-2-deoxy-fluoro-D-glucose (FDG) positron-emission tomography/computed tomography (PET/CT) or locally advanced unresectable primary tumor on contrast-enhanced CT scan, (v) adequate renal, hepatic and hematologic function, including serum creatinine < 1.5 mg/dL, ALT < 2 times upper the limit of the normal range, hemoglobin ≥ 9 g/dL, WBC > 4000/μL, and absolute neutrophil count > 1500/μL. Patients who had received adjuvant or palliative chemotherapy or radiation therapy more than 6 weeks before the start of the study, were also included.

Exclusion criteria were limited to serious cardiovascular problems (*i.e*., heart failure, instable angina, ventricular arrhythmia, ejection fraction < 50%), presence of obstructive jaundice or recent replacement of infected biliary stenting.

There were 29 (78.4%) treatment-naive patients and 8 (21.6%) previously treated patients. Sites of metastases on PET/CT were liver (*N* = 21 out of 31, 67.7%), pancreas (*N* = 19, 61.3%), peritoneum (*N* = 13, 41.9%), regional lymph nodes (*N* = 10, 32.3%), lung (*N* = 6, 19.4%) or other sites (*N* = 3, 9.7%).

### Procedures

NAB-P (125 mg/m^2^) and GEM (1000 mg/m^2^) were administered on day 1, 8, 15 every 28 days for at least 3 cycles. A dose reduction of 25% for patient with ECOG-PS = 2 was planned at the beginning of treatment, followed by dose increase if good tolerance and PS improvement appeared. In the presence of stable disease or objective response detected on CT scan, the treatment continued until 6–7 cycles. In presence of G 3–4 neutropenia, after recovery of toxicity, a secondary prophylaxis with granulocyte-colony stimulating factor (G-CSF) was employed in the next cycles. In patients with a higher degree of toxicity (*G* > 4), recovery to *G* ≤ 1 before continuing therapy was required. Overall, 147 cycles (median 4, range 1–11 cycles) were administered.

### Statistical analysis

Survival probabilities were estimated by means of the Kaplan-Meier method and compared, at univariate analysis, using the log-rank test. Parameters with a statistically significant log-rank test were considered independent variables and included in the multivariate Cox proportional hazard regression linear model to compare hazard ratio (HR) and 95% confidence interval (95% CI). A two-sided *p*-value < 0.05 was considered statistically significant. SAS version 9.20 (SAS Institute Inc., Cary, NC, USA, 2002–2008) was used for statistical analysis.

## CONCLUSION

NAB-P/GEM should be used in all patients with advanced or metastatic PC, with the exception of those with serious contraindications to chemotherapy, such as renal or hepatic impairment or major cardiovascular diseases. Future studies, performed on less selected patient populations, are required to confirm these preliminary outcomes.
